# First Molecular Characterization and Antibiogram of Bacteria Isolated From Dairy Farm Wastewater in Bangladesh

**DOI:** 10.1155/vmi/7253393

**Published:** 2025-05-25

**Authors:** Md. Shamsul Islam, Md. Arif-Uz-Zaman Polash, Md. Hakimul Haque

**Affiliations:** ^1^Department of Veterinary and Animal Sciences, Faculty of Veterinary and Animal Sciences, Rajshahi University, Rajshahi 6205, Bangladesh; ^2^Biomedical Sciences and Molecular Biology, College of Public Health, Medical and Veterinary Sciences, James Cook University, Townsville 4811, Queensland, Australia

**Keywords:** antibiogram, antibiotic resistance, bacteria, Bangladesh, dairy farm wastewater, molecular characterization

## Abstract

This pioneering study in Bangladesh combines phenotypic and genotypic approaches to characterize antibiotic-resistant bacteria in dairy farm wastewater, addressing a critical gap in regional antimicrobial resistance (AMR) research. Dairy farming is integral to global food production, yet the wastewater generated by these operations is a significant source of environmental and public health concerns, particularly in the context of antibiotic resistance. This study aimed to isolate and identify antibiotic-resistant bacteria from dairy farm wastewater and evaluate their antibiogram profiles to inform effective management strategies. A total of 60 wastewater samples were collected and subjected to conventional bacterial characterization, followed by molecular detection via PCR and 16S rRNA gene sequencing. The study identified *Pseudomonas aeruginosa* (35%), *Escherichia coli* (30%), *Bacillus subtilis* (16.67%), and *Acinetobacter junii* (8.33%) as the predominant bacterial species. Sequencing results demonstrated high compatibility with reference sequences, confirming the identities of the isolates. Antibiogram analysis revealed significant resistance patterns: *P. aeruginosa* exhibited the highest resistance to penicillin (85.71%) and amoxicillin (76.19%), while demonstrating greater sensitivity to ciprofloxacin and cotrimoxazole*. E. coli* showed notable resistance to penicillin (88.89%), amoxicillin, and ceftriaxone, while *B. subtilis* and *A. junii* also demonstrated high levels of resistance to multiple antibiotics. Notably, a substantial proportion of the isolates exhibited multidrug resistance (MDR), with MAR indices ranging from 0.37 to 0.75. Moreover, several antibiotic resistance genes (ARGs) including *penA*, *bla*_*TEM*_, *bla*_*CTX*−*M*_, *tetA*, *tetB*, *tetC*, and *ermB* were detected across the bacterial species, with high prevalence rates in *P. aeruginosa* and *A. junii*, suggesting the potential for horizontal gene transfer and further spread of resistance. These findings underscore the critical need for a One Health approach to mitigate the risks posed by antibiotic-resistant bacteria in dairy farm wastewater, emphasizing the critical importance of responsible antibiotic use and sustainable farming practices to protect public health and environmental integrity.

## 1. Introduction

The dairy industry is crucial to the global food system as it provides essential nutrients, especially proteins that are vital for human health and development. In Bangladesh, the dairy sector plays a significant role in the economy and in addressing nutritional deficiencies in the population [1]. Milk and dairy products are rich in high-quality proteins that comprehend all the necessary amino acids for body tissue growth, repair, and maintenance. This makes dairy an indispensable part of the diet for millions of people [2, 3]. However, the rapid growth of the dairy industry has presented notable environmental challenges, particularly concerning wastewater management. Dairy wastewater is a byproduct of dairy processing activities and contains high levels of organic matter, nutrients, and potentially harmful microorganisms [4]. This wastewater can pose severe environmental, animal, and public health risks if not adequately treated. The release of untreated or insufficiently treated dairy wastewater into natural water bodies leads to pollution of surface and groundwater, resulting in eutrophication, aquatic biodiversity loss, and drinking water source contamination [5].

Antimicrobial resistance (AMR) can potentially become the next foremost global health crisis of the 21^st^ century [6]. Globally, AMR already poses a significant threat to humans, animals, and the environment. The improper and excessive use of antibiotics has exerted discerning pressure, resulting in the emergence of multidrug resistance (MDR) in various bacteria [7]. AMR seriously threatens economic growth and human well-being, predominantly in low- and middle-income countries (LMICs) in Asia and Africa. AMR spreads through various pathways, including food chains, the environment, and human and animal waste [8]. We hypothesized that dairy farm wastewater in Bangladesh harbors MDR bacteria with diverse resistant genes, driven by local antibiotic use practices, posing significant risks to public health and environmental health.

Dairy farms possess a highly intricate microbial ecosystem, making dairy wastewater a reservoir for pathogenic and nonpathogenic bacteria. Common bacterial species found in dairy wastewater include *Escherichia coli*, *Salmonella* spp., *Pseudomonas aeruginosa*, *Bacillus subtilis*, *Lactobacillus delbrueckii*, *Enterococcus hirae*, and *Staphylococcus aureus* [4, 9]. A primary concern associated with dairy wastewater is the growth of AMR bacteria. The use of antibiotics in veterinary medicine and the presence of residual antibiotics in dairy products contribute to the development and spread of AMR bacteria in wastewater [10]. Moreover, the horizontal transfer of genes via mobile genetic elements enables these bacteria to develop AMR strains. The nutrient-rich environment of dairy wastewater promotes the proliferation of these bacteria, which can be transmitted to humans through contaminated water, direct contact, or consuming contaminated food products [11]. These bacteria, which have acquired resistance to commonly used antibiotics, pose a significant threat to public health by diminishing the effectiveness of antimicrobial treatments and heightening the danger of infectious disease outbreaks [12, 13].

Given the critical importance of addressing AMR, current research has recently been conducted on humans, domesticated animals, and environmental systems. Despite the widespread prevalence of AMR, much remains unknown about its primary drivers, the role of the natural environment in its spread, and the health risks associated with various environmental and other exposures [14]. In Bangladesh, dairy wastewater management is often inadequate due to limited infrastructure, with untreated effluents frequently discharged into local water bodies, exacerbating AMR risks [15].

While several studies have identified bacterial isolates from dairy farms in Bangladesh, Korea, and Australia [9, 15, 16], none have focused explicitly on detecting bacteria and their resistance genes both phenotypically and genotypically in dairy wastewater in Bangladesh. Therefore, this study aims to detect MDR bacteria and their resistance genes, both phenotypically and genotypically in the wastewater of dairy farms in Bangladesh.

## 2. Materials and Methods

### 2.1. Study Area, Collection, and Transportation of Samples

The Institute of Biological Science (IBSc) at the University of Rajshahi, Bangladesh, granted ethical approval (Memo No. 56/321/IAMEBBC/IBSc) for all research methods and protocols. Sixty samples were collected from dairy farm wastewater across various locations within the Rajshahi City Corporation area of Bangladesh, including 15 samples each from Rajshahi University farm, Narikelbaria farm, Koroitola, and Budhpara. These farms are situated in densely populated regions of the Rajshahi City Corporation. These farms were selected to represent diverse dairy farm practices in Rajshahi City Corporation, varying in scale, management practices, and proximity to urban areas, ensuring a comprehensive assessment of regional AMR patterns. Wastewater samples were aseptically collected in sterilized plastic containers from the waste disposal canals of these dairy farms. The samples were then promptly transported to the Department of Veterinary and Animal Sciences at Rajshahi University for bacteriological analysis. During transportation, stringent measures were implemented to maintain sterile conditions and ensure a cold chain, thereby preserving the samples' integrity for accurate analysis.

### 2.2. Bacterial Isolation and Identification

Initially, a sterile loop full of each sample was inoculated into the nutrient broth. Subsequently, each sample was streaked directly onto chromogenic agar, followed by various selective media, including EMB agar, MacConkey agar, mannitol salt agar, and blood agar base (HiMedia, India) enriched with 7% defibrinated goat blood. These broths and plates were incubated aerobically at 37°C for 24 h to observe specific colony characteristics. Colony counts were performed to assess significant bacterial growth. Discrete colonies were subjected to Gram staining, followed by subculturing to obtain pure cultures. Morphological and biochemical characterization of *P. aeruginosa*, *E. coli*, *B. subtilis*, and *Acinetobacter junii* was carried out using carbohydrate fermentation tests, catalase tests, methyl red tests, Voges–Proskauer tests, indole tests, and reactions in TSI agar, as described by Cheesbrough [17].

### 2.3. Molecular Analysis

#### 2.3.1. Genomic DNA Preparation

We extracted and purified genomic DNA from pure cultures of *P. aeruginosa*, *E. coli*, *B. subtilis*, and *A. junii* using the boiling method outlined by Mahmud et al. [18]. Briefly, 200 μL of deionized ultrapure water was added to an Eppendorf tube. A loop full of pure bacterial colonies from the overnight culture grown at 37°C was introduced into the tube and gently mixed to form a homogeneous cell suspension. The suspension was then boiled for 10 min and chilled for another 10 min. Subsequently, the bacterial cell suspension tubes were centrifuged at 10,000 rpm for 10 min. Each tube collected 100 μL of the supernatant containing bacterial DNA. The purified DNA was assessed for quality and quantity using a NanoDrop Spectrophotometer (BioLab, Ipswich, MA, USA), and the DNA was stored at −20°C until further use.

#### 2.3.2. Polymerase Chain Reaction (PCR) Amplification

Using an established PCR method with specifically designed primers, we identified all isolates as *P. aeruginosa*, *E. coli*, *B. subtilis*, and *A. junii* along with their respective resistance genes, such as those for penicillin (penA), oxytetracycline (tetA, tetB, and tetC), amoxicillin (bla_TEM_), ceftriaxone (bla_CTX-M_), and erythromycin (ermB) (Table 1; Figure S2). The PCR was performed in a final volume of 20 μL, which included 10 μL of PCR Master Mix (Thermo Fisher Scientific, USA), 1 μL of each primer (10 pmol/μL), 1 μL of genomic DNA (50 ng/μL), and 7 μL of nuclease-free water. The amplification products were resolved by electrophoresis using a 1.5% agarose gel. Ethidium bromide was employed for staining, and the bands were visualized under a UV transilluminator (Biometra, Germany). A 1-KB DNA ladder (Thermo Fisher Scientific, MA, USA) was used as a molecular weight reference.

#### 2.3.3. Purification of PCR Products and Sequencing

Successfully amplified specific PCR bands were excised and purified by using the NucleoSpin Gel and PCR Clean-up kit (Macherey-Nagel, Bethlehem, PA, USA) as per the manufacturer's protocols. The purified DNA was then combined with the primer (10–40 ng of DNA) and 1 μL primers (3.2 pmol primers in 10 μL of H_2_O) and subjected to Sanger sequencing for further confirmation of *P. aeruginosa*, *E. coli*, *B. subtilis*, and *A. junii*. Sequencing was executed via an ABI PRISM 3730xl Capillary Sequencer (Applied Biosystems, USA) under standardized PCR cycling conditions and analyzed by MEGA11 software.

#### 2.3.4. Phylogenetic Tree Analysis

The base sequences of the PCR products were compared and aligned with known 16S ribosomal RNA gene sequences from the same genus, which were randomly selected from the GenBank database using multiple sequence alignment. Additionally, the 16S rRNA gene sequences of the isolates from the same species but different countries were referenced, along with their GenBank accession numbers, for phylogenetic analysis, including *Pseudomonas*, *Escherichia*, *Bacillus*, and *Acinetobacter*. The neighbor-joining method was used to determine the evolutionary relationships of the bacterial isolates [23]. In addition, the maximum composite likelihood method was used to compute the evolutionary distances to determine the base substitution number per site [24]. All evolutionary analyses were conducted using MEGA11 software.

### 2.4. Antimicrobial Susceptibility Test

The Kirby–Bauer disk diffusion method [25] was employed to determine the antibiogram phenotype against six commonly used antibiotic classes: penicillins (penicillin 30 μg; amoxicillin 30 μg), cephalosporins (cephradine 25 μg; ceftriaxone 30 μg), macrolides (erythromycin 15 μg), fluoroquinolones (ciprofloxacin 5 μg), tetracycline (oxytetracycline 30 μg), and folate pathway inhibitors (cotrimoxazole: sulfamethoxazole 23.75 μg and trimethoprim 1.25 μg). The selected antibiotics represent classes commonly used in veterinary and human medicine in Bangladesh (penicillins, cephalosporins, macrolides, fluoroquinolones, tetracycline, and folate pathway inhibitors), reflecting local usage patterns and resistance concerns. The antimicrobial susceptibility test was piloted using the disk diffusion method on Mueller–Hinton agar (HiMedia, India) plates with a bacterial concentration equivalent to the 0.5 McFarland standards, followed by aerobic incubation at 37°C for 18–24 h. The characterization as sensitive or resistant is based on the diameters of the zones of inhibition by the results of the antimicrobial susceptibility profiling, ensuing the references of the European Committee on Antimicrobial Susceptibility Test [26]. MDR isolates were classified according to the criteria outlined by Sweeney et al. [27]. Additionally, the multiple antibiotic resistance (MAR) index was considered using the formula: MAR = *a*/*b*, where “*a*” denotes the number of antibiotics to which a specific isolate is resistant and “*b*” denotes the total number of antibiotics examined [28].

### 2.5. Statistical Analysis

We utilized Microsoft Excel 2010 for data entry and IBM SPSS Version 24 (Armonk, NY, USA) for statistical analysis. We determined the rate of occurrence using qualitative statistics. A chi-square (*χ*^2^) test was conducted to evaluate the prevalence of *P. aeruginosa*, *E. coli*, *B. subtilis*, and *A. junii* isolates in various locations within the Rajshahi City co-province area. A *p* value of < 0.05 was estimated as statistically significant.

## 3. Results

### 3.1. Prevalence of Bacteria in Dairy Farm Wastewater

Among the 60 bacteriologically analyzed samples, 54 were clinically suspected to be positive. *P. aeruginosa* was confirmed in 21 samples (35%), *E. coli in* 18 samples (30%), *B. subtilis in* 10 samples (16.67%), and *A. junii in* 5 samples (8.33%), as shown in Table 2. Identification involved isolation on selective media, Gram staining, cultural properties, and biochemical assays. At the Rajshahi University farm, *P. aeruginosa* was most prevalent (8, 53.33%), followed by *E. coli* (5, 33.33%), *A. junii* (3, 20%), and *B. subtilis* (2, 13.33%). In the Narikelbaria dairy farm, *P. aeruginosa* dominated (4, 26.67%), followed by *E. coli* (2, 13.33%) and *B. subtilis* (1, 6.67%); *A. junii* was absent. At Koroitola, *P. aeruginosa* was most frequent (7, 46.67%), followed by *E. coli* (6, 40%), *B. subtilis* (4, 26.67%), and *A. junii* (2, 13.33%). Finally, at Budhpara, *E. coli* was most common (5, 33.33%), followed by *B. subtilis* (3, 20%) and *P. aeruginosa* (2, 13.33%). Overall, *P. aeruginosa* (35%) had a higher occurrence rate than other dairy farm wastewater isolates.

### 3.2. Genetic Detection of Bacteria in Dairy Farm Wastewater by 16S rRNA

All isolates identified through conventional methods were further confirmed using PCR, followed by Sanger sequencing. In these isolates, 1400 base pair fragments were amplified by targeting the 16S rRNA gene, as illustrated in Figure S1. Sanger sequencing data were analyzed using the NCBI BLAST tool, which revealed 97.66% homology for *P. aeruginosa* (accession number PP813651.1), 98.95% homology for *E. coli* (accession number PP817791.1), 97% homology for *B. subtilis* (accession number PP819405.1), and 99.86% homology for *A. junii* (accession number PP819514.1) from the GenBank analysis. These results were consistent with the biochemical findings.

### 3.3. Phylogenetic Analysis

**Figure 1:**
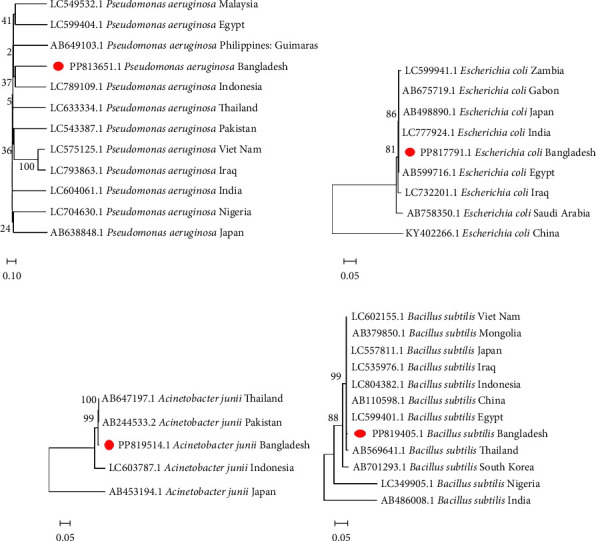
Phylogenetic tree constructed using the 16S ribosomal RNA gene sequences from various bacterial species, including (a) *Pseudomonas aeruginosa*, (b) *E. coli*, (c) *Acinetobacter junii,* and (d) *Bacillus subtilis,* sourced from different countries.

To comprehend the evolutionary relationships, four different phylogenetic trees were constructed for *P. aeruginosa*, *E. coli*, *B. subtilis*, and *A. junii* using 16S rRNA gene sequences including isolates from 12, 9, 12, and 5 countries, respectively.  Figure 1 displays the phylogenetic trees, which illustrates the genetic distances between the Bangladeshi isolates and those from other nations. The alignments showed partial sequence similarity in the 16S ribosomal RNA gene across specific regions. The phylogenetic analysis revealed that the Bangladeshi isolates of *P. aeruginosa*, *E. coli*, *B. subtilis*, and *A. junii* are evolutionarily linked with the other representative isolates of the same species found worldwide.

### 3.4. Antibiogram Profiling

**Figure 2:**
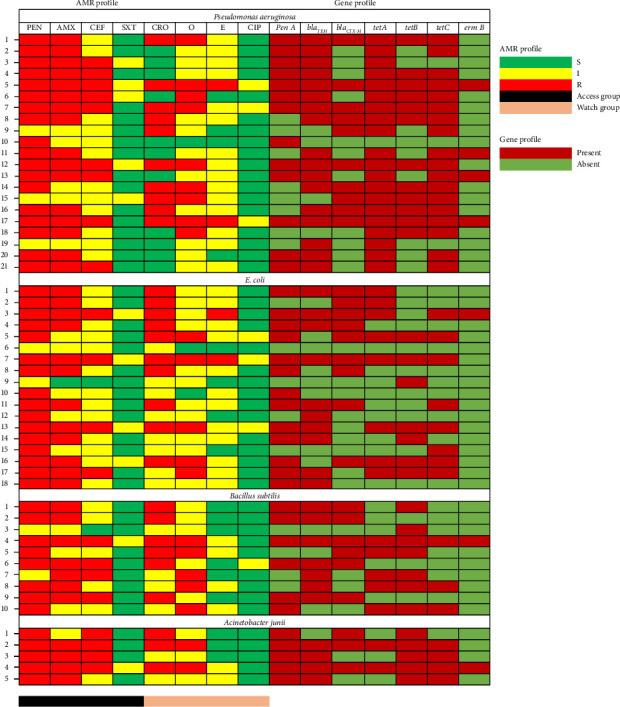
Heat map exhibiting the antibiogram profiles of *Pseudomonas aeruginosa*, *E. coli*, *Bacillus subtilis, Acinetobacter junii* isolated from dairy wastewater samples from the University of Rajshahi, Bangladesh. PEN = penicillin, AMX = amoxicillin, E = erythromycin, CEF = cephradine, CRO = ceftriaxone, SXT = cotrimoxazole, O = oxytetracycline, CIP = ciprofloxacin, S = sensitive, I = intermediate, R = resistant.

The antibiotic susceptibility results indicated that all bacterial isolates exhibited resistance to various antibiotics (Figure 2). For 21 isolates of *P. aeruginosa*, the highest resistance was observed with penicillin (85.71%) and amoxicillin (76.19%), while ciprofloxacin (85.71%) and cotrimoxazole (76.19%) were the most effective. Among the 18 *E. coli* isolates, resistance was highest against penicillin (88.89%), amoxicillin (55.56%), and ceftriaxone (55.56%), whereas ciprofloxacin (83.33%) and cotrimoxazole (77.78%) showed significant effectiveness. For the 10 *B. subtilis* isolates, the highest resistance was to penicillin (80%), followed by amoxicillin (70%), ceftriaxone (60%), and oxytetracycline (50%), while ciprofloxacin and cotrimoxazole demonstrated 90% effectiveness. Lastly, for 5 *A. junii* isolates, penicillin (100%) and both amoxicillin and cephradine had 80% resistance, while ciprofloxacin (100%) and cotrimoxazole (80%) were highly effective.

### 3.5. MDR and MAR Profiles of Dairy Farm Wastewater Isolates

In this study, approximately 33.33% (7 out of 21) of *P. aeruginosa*, 33.33% (6 out of 18) of *E. coli*, 30% (3 out of 10) of *B. subtilis*, and 40% (2 out of 5) of *A. junii* exhibited resistance to more than three classes of antibiotics. Bacteria that are resistant to three or more antibiotics from different classes are classified as MDR. Specifically, 40% of *A. junii*, 33.33% of *P. aeruginosa* and *E. coli*, and 30% of *B. subtilis* demonstrated MDR traits. The antibiotic resistance (AR) profiles of individual *P. aeruginosa* and *E. coli* isolates varied with MAR indices ranging from 0.37 to 0.75. Similarly, *B. subtilis* exhibited MAR indices within the same range, and MDR isolates are detailed in Table 3.

### 3.6. Distribution of AR Genes

In a PCR-based analysis of resistance genes in bacterial isolates, notable variation was observed among different species (Figure 2). For *P. aeruginosa*, the prevalence of resistance genes was as follows: *penA* in 61.90% of isolates, *blaTEM* in 80.95%, *blaCTX-M* in 52.38%, *tetA* in 95.23%, *tetB* in 57.14%, *tetC* in 85.71%, and *ermB* in 19.05%. In *E. coli* isolates, the detected resistance genes included *penA* in 72.22%, *blaTEM* in 55.56%, *blaCTX-M* in 50%, *tetA* in 44.44%, *tetB* in 38.89%, *tetC* in 44.44%, and *ermB* in 5.56%. For *B. subtilis*, resistance gene frequencies were *penA* in 60%, *blaTEM* in 70%, *blaCTX-M* in 60%, *tetA* in 50%, *tetB* in 70%, *tetC* in 30%, and *ermB* in 10%. Finally, in *A. junii* isolates, gene detection rates included *penA* at 100%, *blaTEM* at 80%, *blaCTX-M* at 60%, *tetA* at 40%, *tetB* at 100%, *tetC* at 80%, and *ermB* at 20%. These findings highlight the diversity in resistance profiles across bacterial species, emphasizing the need for tailored antimicrobial strategies based on pathogen-specific resistance patterns.

## 4. Discussion

Dairy farming is a cornerstone of global food production, providing essential commodities such as milk, cheese, and butter. However, this industry also generates substantial quantities of wastewater containing a diverse array of contaminants, including pathogenic microorganisms. A thorough understanding of the microbial composition of dairy farm wastewater is crucial due to its potential implications for public health, environmental sustainability, and the development of antibioticresistance (AR) [29].

This study revealed the significant prevalence of MDR bacteria in dairy farm wastewater, with *P. aeruginosa*, *E. coli*, *B. subtilis*, and *A. junii* being the most frequently isolated species. Notably, *P. aeruginosa* was identified in 35% of the samples, making it the predominant isolate, followed by *E. coli* at 30%. These findings are aligned with prior research conducted in agricultural settings, where *P. aeruginosa* has demonstrated remarkable adaptability and resilience in harsh environmental conditions due to its biofilm-forming capabilities [29–31]. Although *E. coli* was less prevalent, its presence is notable as it serves as a fecal contamination indicator and poses a potential health risk. Our finding aligns with regional studies in Bangladesh, such as Rezwana et al. [15], who reported higher bacterial load in dairy wastewater, and in India, Soni et al. [32], who reported similar MDR patterns in wastewater, suggesting shared environmental and agricultural drivers of AMR in South Asia. Another study conducted in Australia similarly identified *Bacillus cereus* and *Clostridium perfringens* as the dominant pathogens in dairy wastewater, with prevalence of 41% and 38%, respectively, suggesting variations influenced by local ecological factors and farm management practices [16].

Genetic identification using 16S rRNA sequencing confirmed the accuracy of traditional biochemical identification methods, showing high sequence homology with known strains. Phylogenetic analysis further revealed evolutionary relationships, indicating potential horizontal gene transfer and the sharing of resistance mechanisms, likely driven by agricultural practices and environmental pressures [33, 34]. Phylogenetic similarities with global isolates suggest potential horizontal gene transfer of resistance genes, highlighting the need for regional and global AMR surveillance to track gene dissemination.

The emergence of AMR poses a significant global challenge, particularly in Bangladesh, where it has profound implications for public health and healthcare systems [35]. Antibiotic residues in wastewater likely stem from unregulated use in dairy farming, where antibiotics such as penicillin and tetracycline are routinely administered for prophylaxis, contributing to resistance development [34]. In this study, antibiotic susceptibility testing revealed high resistance rates among *P. aeruginosa* and *E. coli* to commonly used antibiotics such as penicillin and amoxicillin. These resistance patterns are consistent with previous studies on the dairy and aquaculture industries, highlighting the potential misuse of antibiotics in animal farming as a driving factor for AMR [36–38]. Soil-borne bacteria such as *B. subtilis* also exhibited resistance to amoxicillin and ceftriaxone, likely due to environmental antibiotic exposure [39]. *A. junii*, another notable MDR species, showed varying resistance levels, reflecting the broader global concern regarding environmental bacteria's capacity to develop resistance [40]. Encouragingly, ciprofloxacin and cotrimoxazole demonstrated significant efficacy against these isolates, suggesting their potential use as alternative treatment options in regions with high AMR prevalence. The calculated MAR indices ranged from 0.37 to 0.75 across species, underscoring frequent antibiotic exposure in the study area and corroborating concerns about the amplification of resistance due to unregulated antimicrobial usage [29].

The detection of resistance genes, including *blaTEM*, *blaCTX-M*, and *tetA*, highlights the risk of horizontal gene transfer in wastewater environments, which could facilitate the spread of resistance genes to other microbial communities, including human pathogens [32, 41]. These findings align with previous research emphasizing wastewater as a key reservoir for AR genes (ARGs) and their dissemination across diverse bacterial species [42, 43]. The high frequency of MDR strains, including 40% among *A. junii*, poses a significant public health concern, particularly given this bacterium's association with nosocomial infections and its ability to persist in aquatic environments. The detection of MDR strains in 33.33% of *P. aeruginosa* and *E. coli* isolates further underscores the critical public health and environmental risks posed by dairy wastewater, including the potential spread of AMR through water systems. This scenario highlights the need for improved wastewater management and stricter regulatory frameworks to reduce AMR dissemination. Addressing this issue requires a holistic “One Health” approach, emphasizing the interconnectedness of human, animal, and environmental health. To mitigate AMR risks, we recommend adopting advanced wastewater treatment technologies, such as anaerobic digestion, reducing bacterial loads and ARGs, alongside enforcing antibiotic stewardship programs in dairy farming to limit unnecessary antibiotic use, aligning with Bangladesh's National Action Plan on AMR. This study was limited to Rajshahi City Corporation, potentially underrepresenting Bangladesh's diverse dairy practices. Future research should encompass multiple regions and longitudinal sampling to capture seasonal and temporal AMR trends.

## 5. Conclusion

This study for the first time provides a comprehensive analysis of AMR among *P. aeruginosa*, *E. coli*, *B. subtilis*, and *A. junii* isolates from dairy farm wastewater, shedding light on resistance profiles and sensitivity patterns that underscore the complexity of AMR in agricultural environments. The high resistance rates observed, especially to penicillin, amoxicillin, cephradine, ceftriaxone, and oxytetracycline, highlight the significant prevalence of MDR bacteria, which could complicate therapeutic interventions for infections associated with these pathogens. Sensitivity to ciprofloxacin and cotrimoxazole suggests that these antibiotics may remain effective treatment options; however, their usage should be managed carefully to prevent further resistance. The study underscores the need for a multifaceted approach to tackle AMR, emphasizing coordinated actions such as rigorous biosecurity, strict hygiene practices, advanced wastewater treatment, and the rational use of antibiotics in livestock management. The findings stress that local AMR surveillance is essential to inform effective antibiotic stewardship and guide appropriate prescribing practices tailored to regional resistance patterns. Addressing AMR in Bangladesh requires ongoing research to monitor resistance trends, understand the role of horizontal gene transfer in spreading resistance, and develop context-specific strategies for mitigating AMR. Comprehensive surveillance programs, public health policies, and stakeholder collaboration are critical for combating the global threat of AMR and ensuring the sustainability of antimicrobial treatments in healthcare and agriculture.

## Figures and Tables

**Table 1:** List of primers for the detection of bacteria and their resistance genes from dairy wastewater.

Target gene	Primer sequence (5′–3′)	Product size (bp)	Annealing temperature (°C)	Reference
16S rRNA	27 F: AGAGTTTGATCCTGGCTCAG	1400	48	[19]
	1492 R: CGGTTACCTTGTTACGACTT			

*penA*	PenA F: ATCGAACAGGCGACGATGTC	500	65	[20]
	PenA R: GATTAAGACGGTGTTTTACGG			

*bla*_*TEM*_	blaTEM F: GCGGAACCCCTATTTG	964	50	[21]
	blaTEM R: TCTAAAGTATATATGAGTAAACTTGGTCTGAC			

*bla*_*CTX*−*M*_	CTXM F: ACGCTGTTGTTAGGAAGTG	857	58	[22]
	CTXM R: TTGAGGCTGGGTGAAGT			

*tetA*	TetA F: GGCGGTCTTCTTCATCATGC	502	64	[20]
	TetA R: CGGCAGGCAGAGCAAGTAGA			

*tetB*	TetB F: GAGACGCAATCGAATTCGG	228	58	[20]
	TetB R: TTTAGTGGCTATTCTTCCTGCC			

*tetC*	TetC F: TGCTCAACGGCCTCAACC	379	58	[20]
	TetC R: AGCAAGACGTAGCCCAGCG			

*ermB*	ErmB F: GAAAAGGTACTCAACCAAATA	639	58	[20]
	ErmB R: AGTAACGGTACTTAAATTGTTTAC			

**Table 2:** Prevalence of *Pseudomonas aeruginosa*, *E. coli*, *Bacillus subtilis*, and *Acinetobacter junii* in different wastewater sample of Rajshahi University regions.

Isolates	Rajshahi University farm	Narikelbaria farm	Koroitola	Budhpara	Total	Chi-square	*p* value
*Pseudomonas aeruginosa*	8 (53.33%)	4 (26.67%)	7 (46.67%)	2 (13.33%)	21 (35%)	7.061	0.070
*E. coli*	5 (33.33%)	2 (13.33%)	6 (40%)	5 (33.33%)	18 (30%)	3.142	0.370
*Bacillus subtilis*	2 (13.33%)	1 (6.67%)	4 (26.67%)	3 (20%)	10 (16.67%)	2.530	0.474
*Acinetobacter junii*	3 (20%)	0 (0%)	2 (13.33%)	0 (0%)	5 (8.33%)	8.180	0.042

**Table 3:** MDR *Pseudomonas aeruginosa*, *E. coli*, *Bacillus subtilis*, and *Acinetobacter junii* isolated from dairy farm wastewater.

Name of bacteria	No. of patterns	Antibiotic resistance patterns	No. of isolates	Overall MDR isolates (%)	MAR index
*Pseudomonas aeruginosa*	1	PEN, AMX, CEF, CRO, E, O	2	7/21 (33.33%)	0.75
	2	PEN, AMX, CEF, CRO, O	2		0.62
	3	PEN, AMX, CRO, O	1		0.50
	4	PEN, AMX, CEF, O	1		0.50
	5	PEN, CRO, O	1		0.37

*E. coli*	1	PEN, AMX, CEF, CRO, E, O	1	6/18 (33.33%)	0.75
	2	PEN, AMX, CEF, CRO, E	1		0.62
	3	PEN, AMX, CEF, CRO, O	1		0.62
	4	PEN, AMX, CRO, O	1		0.50
	5	PEN, AMX, CEF, O	1		0.50
	6	PEN, CRO, O	1		0.37

*Bacillus subtilis*	1	PEN, AMX, CEF, CRO, O	1	3/10 (30%)	0.62
	2	PEN, CRO, O	1		0.37
	3	AMX, CEF, O	1		0.37

*Acinetobacter junii*	1	PEN, AMX, CEF, CRO, O	2	2/5 (40%)	0.62

Abbreviations: AMX = amoxicillin, CEF = cephradine, CIP = ciprofloxacin, CRO = ceftriaxone, E = erythromycin, *E. coli* = *Escherichia coli*, MDR = multidrug-resistant, MAR = multiple antibiotic resistance, O = oxytetracycline, PEN = penicillin, SXT = cotrimoxazole.

## Data Availability

The datasets generated or analyzed during this study are available from the corresponding author upon reasonable request.

## Supporting Information

Figure S1: Gel electrophoresis of *Pseudomonas aeruginosa* (5), *Escherichia coli* (4), *Bacillus subtilis* (3), and *Acinetobacter junii* (2), showing 1400 base pair fragments amplified by targeting the 16S rRNA gene. A 1-kb DNA ladder (1) was used as a molecular weight marker for comparison.

Figure S2: PCR amplification products of selected resistance genes, including (A) *penA*, (B) *blaTEM*, (C) *blaCTX-M*, (D) *tetA*, (E) *tetB*, (F) *tetC*, and (G) *erm*, detected in *Pseudomonas aeruginosa*, *Escherichia coli*, *Bacillus subtilis*, and *Acinetobacter junii*.
