# Supplementary material for: First Molecular Characterization and Antibiogram of Bacteria Isolated From Dairy Farm Wastewater in Bangladesh
Source: Vet Med Int. 2025 May 25;2025:7253393. doi: 10.1155/vmi/7253393 (PMC12127123; doi:10.1155/vmi/7253393)
Supplement: Supporting Information — Additional supporting information can be found online in the Supporting Information section. [file 7253393.f1.docx]

**Supplementary File**

**for**

**First Molecular Characterization and Antibiogram of Bacteria Isolated from Dairy Farm Wastewater in Bangladesh**

Md. Shamsul Islam^1^, Md. Arif-Uz-Zaman Polash^1^, Md. Hakimul Haque^1,2^**^*^**


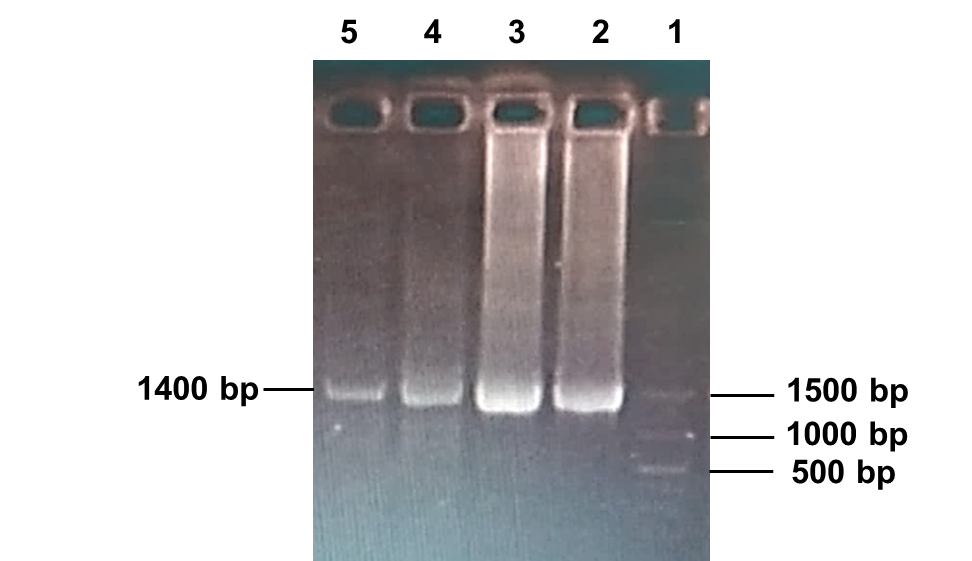


**FIGURE S1**: Gel electrophoresis of *Pseudomonas aeruginosa* (5), *Escherichia coli* (4), *Bacillus subtilis* (3), and *Acinetobacter junii* (2), showing 1400 base pair fragments amplified by targeting the 16S rRNA gene. A 1 kb DNA ladder (1) was used as a molecular weight marker for comparison.

**
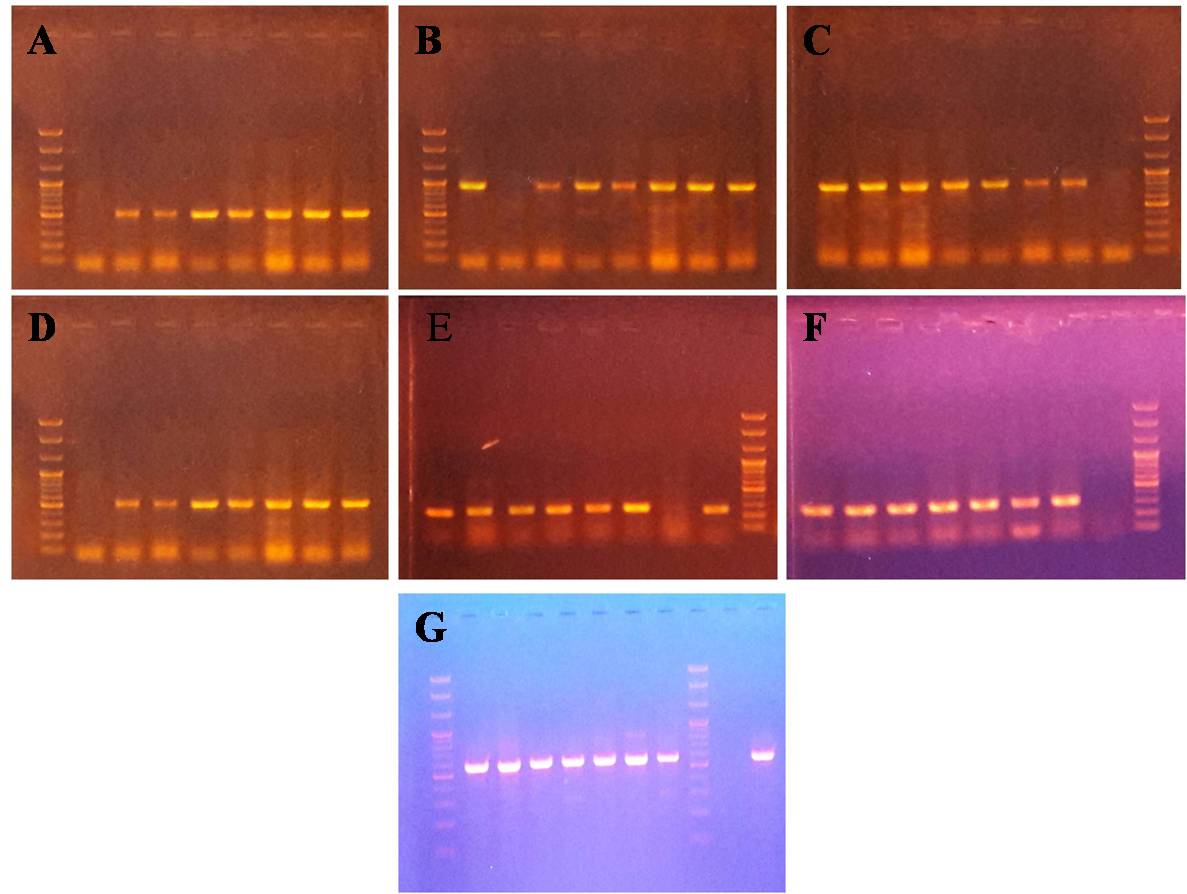
**

**FIGURE S2**: PCR amplification products of selected resistance genes, including (A) *penA*, (B) *blaTEM*, (C) *blaCTX-M*, (D) *tetA*, (E) *tetB*, (F) *tetC*, and (G) *erm*, detected in *Pseudomonas aeruginosa*, *Escherichia coli*, *Bacillus subtilis*, and *Acinetobacter junii*.
